# A lung ultrasound B-line score to stratify oxygen therapy in transient tachypnea of the neonate: a prospective cohort study

**DOI:** 10.7717/peerj.21559

**Published:** 2026-07-22

**Authors:** Kunpeng Liu, Zhirong He, Mimi Zou, Xiaoli Wu, Lin Gan, Lvxia Tang, Xuelian Yan, Yuanchun Chen

**Affiliations:** Chongqing University Fuling Hospital, Chongqing, China

**Keywords:** Lung ultrasound, B-line score, Transient tachypnea of the neonate, Point-of-care ultrasound, Prognostic biomarker

## Abstract

**Background:**

Transient tachypnea of the neonate (TTN) is the most common cause of respiratory distress in newborns. While often self-limiting, the severity of TTN varies significantly, ranging from mild tachypnea to severe respiratory failure requiring non-invasive ventilation. Early and objective grading of TTN severity remains difficult in routine practice. Clinical scores are subjective, and chest radiography is insensitive to the extent of lung fluid. This study evaluated a semi-quantitative lung ultrasound (LUS) B-line score as a predictor of oxygen requirement in neonates with TTN.

**Methods:**

In a prospective cohort at a tertiary neonatal intensive care unit (NICU), we enrolled 267 neonates (gestational age ≥ 33 weeks) with a clinical diagnosis of TTN. Within 6 hours of admission, we recorded a standardized twelve-zone LUS score, respiratory rate, and arterial blood gas values. The primary outcome was the highest level of respiratory support within 72 hours, classified as no oxygen support, low-flow oxygen support, or escalated respiratory support. Analyses included Spearman correlation, receiver-operating-characteristic (ROC) curves, and multivariable ordinal logistic regression.

**Results:**

The LUS score correlated inversely with arterial oxygen (PaO_2_) (*ρ* = −0.705, *P* < 0.001) and positively with partial pressure of arterial carbon dioxide (PaCO_2_) (*ρ* = 0.399, *P* < 0.001). Mean scores rose with increasing support (0.5 ± 2.1, 15.0 ± 6.4, and 27.0 ± 4.8 for room air, low-flow, and high-flow groups; *P* < 0.001). For predicting any oxygen use, the area under the curve (AUC) was 0.982 (95% CI [0.968–0.997]) with an optimal cutoff of 5.5. A cutoff of 22.5 identified infants needing high-flow support (AUC 0.965). In multivariable analysis, the LUS score was the strongest independent predictor of respiratory support level (adjusted odds ratio per point, 1.70; 95% CI [1.41–2.05]; *P* < 0.001).

**Conclusions:**

A semi-quantitative LUS score provides a non-invasive and physiologically coherent measure of disease severity in TTN and can help stratify early oxygen and respiratory support requirements at the bedside. These findings suggest that incorporating LUS scoring into early NICU assessment may help standardize decisions about initiation and escalation of respiratory support in infants with TTN.

## Introduction

Transient tachypnea of the neonate (TTN) is the leading cause of admission to the neonatal intensive care unit (NICU) among term and late-preterm infants. It results from delayed clearance of fetal lung fluid, leading to transient pulmonary edema (Edwards, Kotecha & Kotecha, 2013; Hermansen & Mahajan, 2015; Patti et al., 2025). Although TTN is generally considered a benign and self-limiting condition, the clinical spectrum is variable, ranging from mild tachypnea requiring only observation to more severe respiratory distress necessitating supplemental oxygen, continuous positive airway pressure (CPAP), or, in a minority of cases, invasive mechanical ventilation (Corsini et al., 2019; Hermansen & Mahajan, 2015).

A key challenge in managing TTN is rapidly determining the severity of the condition to provide the appropriate level of intervention. Clinicians must balance the risks of overtreatment against the dangers of hypoxemia and fatigue inherent in delayed support. Current strategies for risk stratification rely on physical examination scores (*e.g.*, Silverman-Andersen score) and chest radiography (CXR). However, clinical scores are subjective and demonstrate significant inter-observer variability (Hermansen & Mahajan, 2015). CXR, the historical standard, exposes infants to ionizing radiation and provides a static, two-dimensional representation. Crucially, radiographic findings in TTN often lag behind physiological changes and correlate poorly with the degree of gas exchange impairment (Corsini et al., 2019; Escourrou & De Luca, 2016; Perri et al., 2018). The white lung appearance is a qualitative finding that defies precise measurement, leaving the clinician to guess the true extent of interstitial edema (De Luca et al., 2017).

Lung ultrasound (LUS) has been established as a reliable, non-invasive imaging modality for the bedside assessment of neonatal lung pathology (Kelner et al., 2026; Kurepa et al., 2018; Lichtenstein & Mezière, 2008). In the context of TTN, the interaction between the ultrasound beam and subpleural fluid-filled interlobular septa generates vertical reverberation artifacts known as B-lines (Volpicelli et al., 2012). The density of these B-lines is directly proportional to the loss of lung aeration (Hiles et al., 2017; Razak & Faden, 2020). While the diagnostic accuracy of LUS for identifying TTN and neonatal respiratory distress syndrome (NRDS) is well-documented, its application has remained largely qualitative (presence *vs.* absence of pathology) rather than quantitative (Liu et al., 2015).

We postulated that if the burden of B-lines reflects the degree of lung aeration loss-which in TTN is primarily attributable to delayed fetal lung fluid clearance-a systematic scoring system should correlate with physiological gas exchange impairment and, by extension, the requirement for respiratory support. Although preliminary scoring systems have been proposed (Copetti & Cattarossi, 2007; Gargani & Volpicelli, 2014), few have been rigorously validated against arterial blood gas (ABG) indices or calibrated to predict specific thresholds of oxygen therapy (Brat et al., 2015; Razak & Faden, 2020; Vergine et al., 2014).

In this prospective cohort study, we developed and applied a study-specific semi-quantitative LUS B-line scoring scheme based on the standard 12-zone neonatal lung ultrasound framework. Our primary objectives were to quantify the relationship between this score and arterial oxygenation (PaO_2_) and ventilation (PaCO_2_), and to explore score thresholds that discriminate between neonates requiring no respiratory support, low-flow oxygen, and escalated respiratory support.

## Methods

### Study design and oversight

We conducted this prospective observational study in the Level III Neonatal Intensive Care Unit at Chongqing University Fuling Hospital from January 1, 2022, to September 30, 2024. The protocol adhered to the principles of the Declaration of Helsinki and was approved by the Institutional Review Board of Chongqing University Fuling Hospital (Ethics Approval Number: 2025CDFSFLYYEC-82). Because the study was observational in nature, did not modify routine clinical management, and all procedures involved-including lung ultrasound examination, arterial blood gas sampling, and respiratory support management, were part of standard neonatal care, the requirement for written informed parental consent was waived by the Institutional Review Board.

### Study population

Eligible participants were neonates with a gestational age of 33 weeks or greater and a birth weight of at least 1,800 g, admitted to the NICU within 24 h of birth. Inclusion required the onset of respiratory symptoms (tachypnea > 60 breaths/minute, grunting, nasal flaring, or retractions) within the first 6 h of life and a preliminary clinical diagnosis of TTN. TTN was diagnosed by the attending neonatology team based on: (1) onset of respiratory distress within the first hours after birth; (2) a clinical course compatible with delayed lung fluid clearance, typically with spontaneous improvement within 24–72 h; and (3) exclusion of alternative diagnoses (including NRDS, pneumonia, meconium aspiration, pneumothorax, and congenital heart disease) through serial clinical assessment, laboratory testing, and imaging when indicated. LUS findings were recorded for study purposes but were not used as the sole determinant of the TTN diagnosis.

We excluded infants with: (1) major congenital anomalies; (2) confirmed meconium aspiration syndrome; (3) pneumothorax; (4) severe perinatal asphyxia (5-minute Apgar score ≤5 or pH <7.0); (5) clinical or radiographic evidence more consistent with NRDS than TTN, based on persistent or worsening oxygen requirement inconsistent with the expected course of TTN, chest radiographic findings suggesting diffuse surfactant deficiency (reticulogranular or ground-glass opacities) rather than patterns typical of transient fluid retention, and/or clinical need for surfactant replacement therapy. Because TTN and mild NRDS may overlap in the early postnatal period, final eligibility was determined after review of the clinical evolution over 24–72 h rather than on a single blood gas result; and (6) early-onset sepsis manifesting as shock, to avoid confounding pulmonary edema with capillary leak syndrome.

### Clinical and physiological assessment

Upon admission, all neonates underwent a standardized clinical evaluation. Respiratory rate and heart rate were recorded continuously. Arterial blood gas (ABG) analysis was performed *via* radial artery puncture or indwelling arterial catheter within 6 h of admission to measure partial pressure of oxygen (PaO_2_), partial pressure of carbon dioxide (PaCO_2_), and pH. The fraction of inspired oxygen (FiO_2_) at the time of blood gas sampling was also recorded, and the PaO_2_/FiO_2_ ratio was calculated. In our NICU, ABG analysis is part of routine clinical care for neonates admitted with clinically significant respiratory distress and was not mandated solely for research purposes. Sampling was performed using standard NICU procedures, with routine non-pharmacological comfort measures according to unit practice and post-procedure monitoring for local bleeding, hematoma, and distal perfusion. No major adverse events attributable to arterial sampling were documented in the medical records during the study period. The decision to initiate, escalate, or wean respiratory support was made by the attending neonatologists according to unit protocols, strictly blinded to the LUS scores.

### Lung ultrasound protocol

LUS examinations were performed within 6 h of admission (concurrent with the physiological stabilization period) by designated sonographers or neonatologists with advanced training in point-of-care ultrasound. For each zone, static images and a cine clip of approximately 3–6 s, including at least one complete respiratory cycle when feasible, were recorded and archived for review. Operators were blinded to the ABG results and the specific clinical grading of TTN severity and were not involved in clinical treatment decisions. LUS scores were recorded on dedicated study forms that were not accessible to the treating clinical team. Conversely, the attending neonatologists made all respiratory support decisions according to routine clinical assessment and unit protocols without knowledge of the LUS scores.

We utilized a high-frequency linear transducer (8–12 MHz) with the infant in the supine position. A twelve-zone scanning protocol was employed. Each lung was longitudinally divided into anterior, lateral, and posterior regions by the anterior and posterior axillary lines. These regions were further divided into upper and lower fields by the nipple line level, resulting in six zones per side and twelve zones in total.

### Scoring system

In each zone, the worst ultrasound pattern observed was scored semi-quantitatively based on the density of B-lines (Copetti & Cattarossi, 2007; Gargani & Volpicelli, 2014). The scoring scheme used in this study was developed on the basis of the conventional 12-zone neonatal lung ultrasound framework and adapted for study-specific semi-quantitative assessment in TTN. Each zone was scored from 0 to 3, where 0 indicated normal A-line pattern with <3 B-lines, 1 indicated ≥3 well-separated B-lines, 2 indicated coalescent B-lines with partial white-lung appearance, and 3 indicated severe coalescent B-lines with diffuse white lung. The total LUS score ranged from 0 to 36, calculated as the sum of scores across all twelve lung zones.

### Outcome definitions

The primary outcome was the maximum level of respiratory support required within 72 h of life, defined as an ordered categorical variable: Level 0 (No oxygen support) indicating room air; Level 1 (Low-flow oxygen support) indicating requirement for low-flow nasal cannula (flow ≤ 2 L/min) or oxygen hood; and Level 2 (Escalated respiratory support) indicating requirement for high-flow nasal cannula (flow>2 L/min), CPAP, or invasive mechanical ventilation. These modalities were combined into a single escalation category because the number of infants requiring advanced support was small, and the primary clinical aim was to identify infants at risk of escalation beyond low-flow oxygen rather than to distinguish between specific ventilatory modalities. Secondary outcomes included the diagnostic accuracy of the LUS score for predicting any oxygen requirement and escalated respiratory support, and the correlations between the LUS score and physiological variables, including PaO_2_, PaCO_2_, pH, respiratory rate, and PaO_2_/FiO_2_ ratio

### Statistical analysis

We estimated that a sample size of 240 infants would provide 90% power to detect an area under the ROC curve (AUC) of 0.85 or greater, assuming a prevalence of oxygen requirement of 60% and a two-sided alpha of 0.05.

Continuous variables were expressed as means ± standard deviations (SD) or medians with interquartile ranges (IQR), depending on distribution. Categorical variables were reported as frequencies and percentages. Differences across the three oxygen therapy groups were assessed using the Kruskal-Wallis test or one-way ANOVA for continuous variables and the Chi-square test for categorical variables.

Correlations between the LUS score and physiological parameters were analyzed using Spearman’s rank correlation coefficient (*ρ*). Receiver operating characteristic (ROC) curves were generated to evaluate the diagnostic performance of the LUS score. Optimal cutoffs were derived using the Youden index (sensitivity+specificity - 1).

To isolate the predictive value of the LUS score from potential confounders, we constructed a multivariable ordinal logistic regression model. Covariates were selected a priori based on clinical plausibility and their potential to confound the relationship between LUS score and respiratory support requirement in TTN. These included gestational age (reflecting lung maturity), mode of delivery (a known risk factor for TTN), PaO_2_, and PaCO_2_ (reflecting contemporaneous gas-exchange status). Model performance was assessed using discrimination (C-statistic) and calibration (bootstrap validation). Decision curve analysis (DCA) was performed to quantify the net clinical benefit of LUS-guided stratification (Vickers & Elkin, 2006). To estimate model optimism, bootstrap internal validation was performed with 200 resamples, and model discrimination and calibration were examined in the resampled datasets. All analyses were conducted using R software, version 4.4.3.

## Results

A total of 267 neonates with TTN were included in the final analysis. No enrolled infants were excluded after baseline assessment, and there were no missing data for the primary predictor or primary outcome. Of these, 85 (31.8%) required no oxygen support, 164 (61.4%) required low-flow oxygen support, and 18 (6.7%) required escalated respiratory support. Baseline characteristics are presented in Table 1. Neonates requiring higher respiratory support were generally of lower gestational age and birth weight and had lower 5-minute Apgar scores (all *P* < 0.001). Detailed demographic and clinical data are provided in Table 1.

**Table 1:** Demographic and clinical characteristics of the study population stratified by maximum oxygen therapy requirement.

**Characteristic**	**Overall** **(*n* = 267)**	**No oxygen support** **(*n* = 85)**	**Low-flow oxygen support (*n* = 164)**	**Escalated respiratory support (*n* = 18)**	**P value**
n	267	85	164	18	
Gestational_Age_weeks (mean (SD))	36.02 (2.16)	37.69 (1.70)	35.24 (1.88)	35.28 (2.03)	<0.001
Weight (mean (SD))	2,536.40 (599.73)	2,970.02 (587.06)	2,328.58 (475.15)	2,382.22 (615.06)	<0.001
Sex = Male (%)	152 (56.9)	49 (57.6)	89 (54.3)	14 (77.8)	0.159
Delivery_Mode = Vaginal (%)	188 (70.4)	53 (62.4)	121 (73.8)	14 (77.8)	0.135
Apgar_1min (mean (SD))	9.32 (1.41)	9.88 (0.39)	9.26 (1.35)	7.17 (2.57)	<0.001
Apgar_5min (mean (SD))	9.71 (0.98)	10.00 (0.00)	9.76 (0.78)	7.89 (2.30)	<0.001
Term_Status = Term (%)	84 (31.5)	54 (63.5)	26 (15.9)	4 (22.2)	<0.001
Meconium_Staining = Yes (%)	25 (9.4)	8 (9.4)	16 (9.8)	1 (5.6)	0.845
Resp_Rate (mean (SD))	55.64 (6.20)	51.80 (5.45)	57.44 (5.10)	57.44 (9.88)	<0.001
PaO2 (mean (SD))	63.54 (19.27)	84.89 (9.74)	55.42 (12.66)	36.67 (11.03)	<0.001
PaCO2 (mean (SD))	37.86 (10.43)	33.20 (6.90)	38.37 (9.47)	55.22 (13.26)	<0.001
PH (mean (SD))	7.35 (0.08)	7.39 (0.07)	7.33 (0.08)	7.31 (0.13)	<0.001
LUS_Score (median [IQR])	12.00 [0.00, 19.00]	0.00 [0.00, 0.00]	16.00 [10.00, 20.00]	27.50 [24.25, 29.00]	<0.001
Resp_Support_Level (%)					<0.001
No Support	85 (31.8)	85 (100.0)	0 (0.0)	0 (0.0)	
Minimal	34 (12.7)	0 (0.0)	34 (20.7)	0 (0.0)	
Moderate	130 (48.7)	0 (0.0)	130 (79.3)	0 (0.0)	
Advanced	18 (6.7)	0 (0.0)	0 (0.0)	18 (100.0)	
Vent_Duration_days (median [IQR])	3.00 [0.00, 9.00]	0.00 [0.00, 0.00]	5.00 [3.00, 12.00]	15.00 [8.25, 24.00]	<0.001
LOS_Days (median [IQR])	9.00 [6.00, 21.00]	6.00 [5.00, 7.00]	14.00 [8.00, 26.00]	24.00 [10.50, 37.50]	<0.001

**Notes.**

Data: mean ± SD, median [IQR], or n (%). *P* values from Kruskal–Wallis H test, ANOVA, or Chi-square test.

### LUS score distribution and physiological parameters

**Figure 1:**
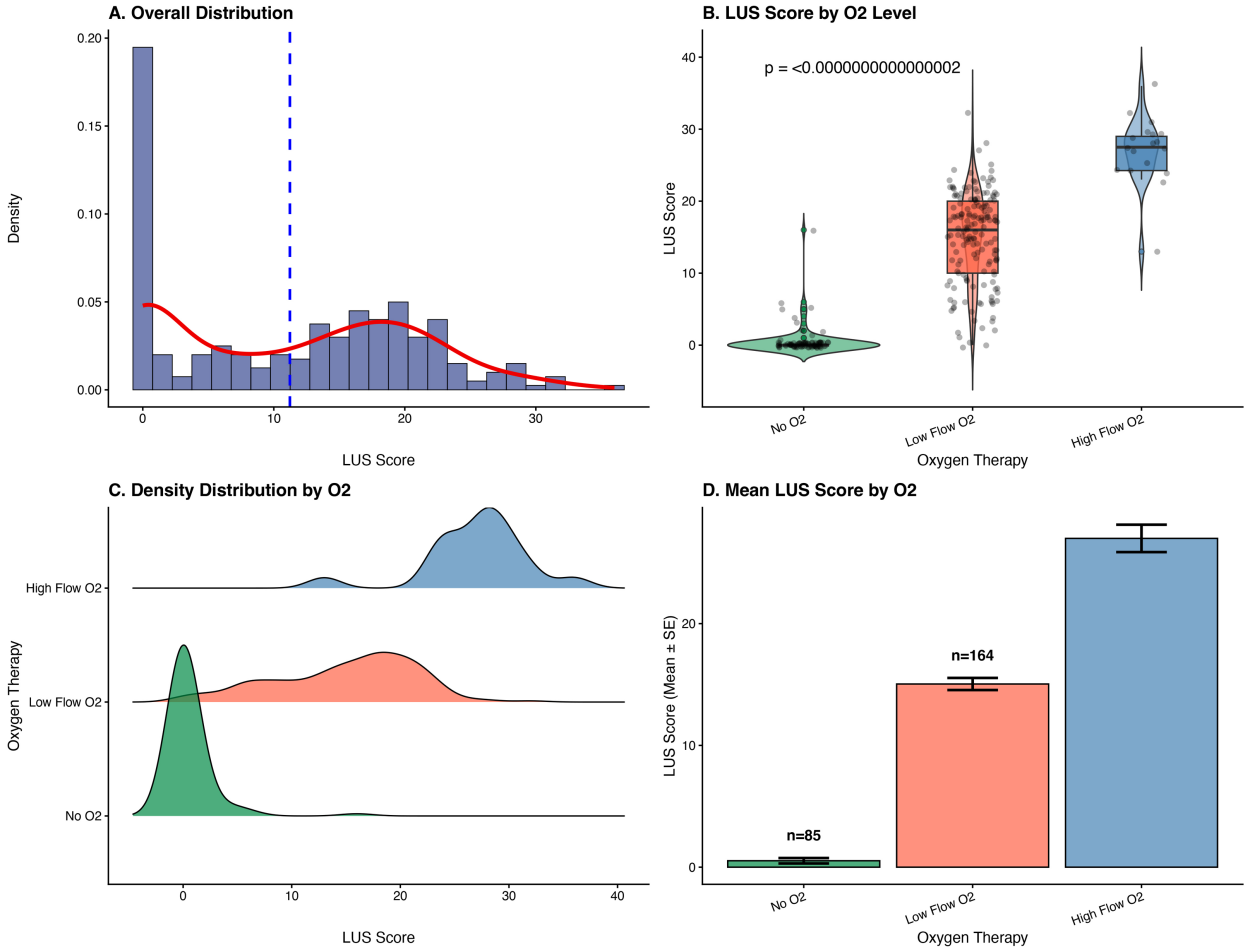
Distribution of LUS score according to maximum respiratory support level.

The LUS score showed a clear stepwise increase across respiratory support levels (Fig. 1). Median LUS scores were 0.0 [0.0–0.0] in the no-support group, 16.0 [10.0–20.0] in the low-flow group, and 27.5 [24.3–29.0] in the escalated-support group (*P* < 0.001; corresponding means: 0.5 ± 2.1, 15.0 ± 6.4, and 27.0 ± 4.8, respectively).

**Figure 2:**
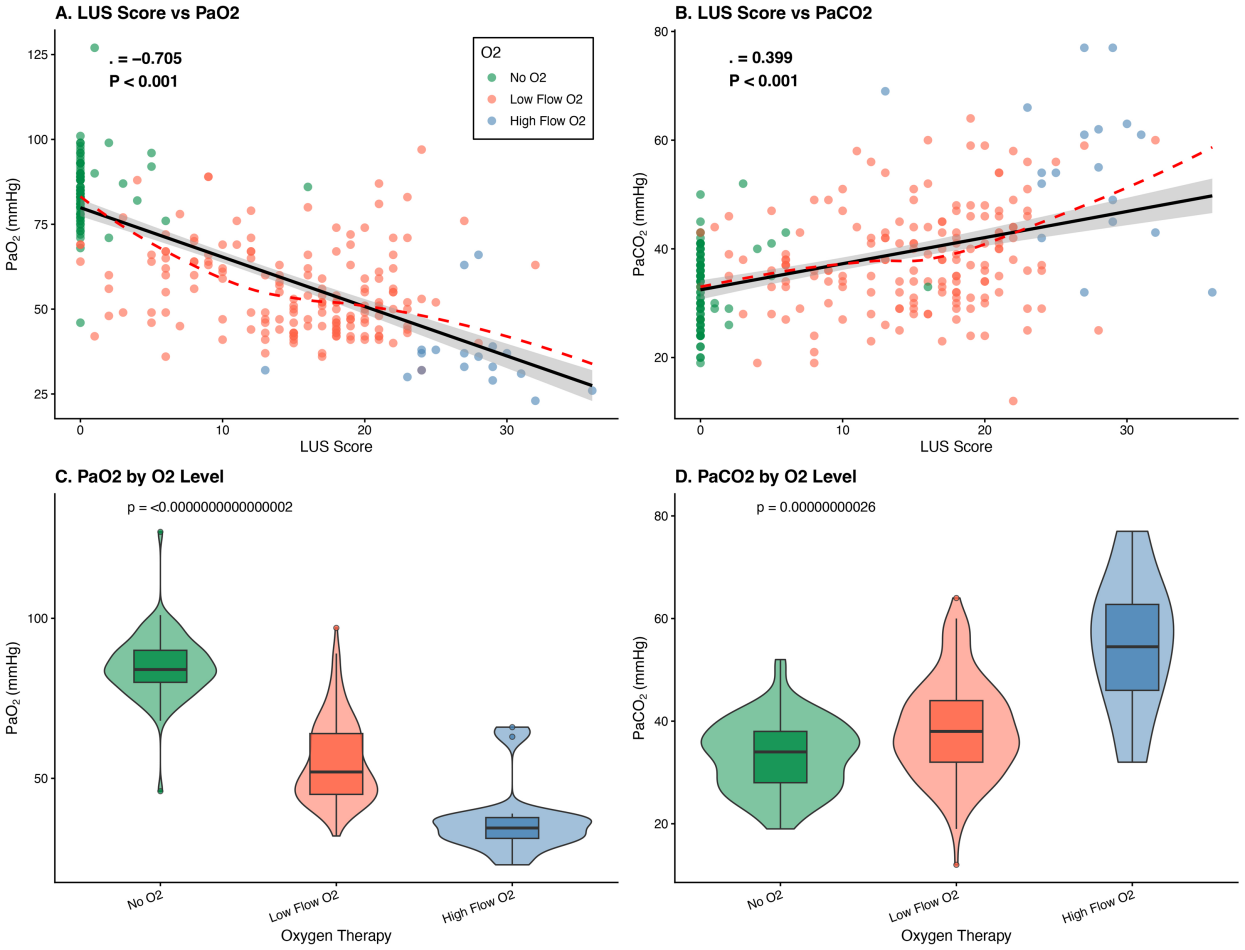
Correlations between LUS score and physiological parameters.

Physiological parameters stratified clearly by severity (Table 2, Fig. 2). Mean PaO_2_ decreased from 84.9 ± 9.7 mmHg to 55.4 ± 12.7 mmHg to 36.7 ± 11.0 mmHg across groups (*P* < 0.001), whereas mean PaCO_2_ increased from 33.2 ± 6.9 mmHg to 38.4 ± 9.5 mmHg to 55.2 ± 13.3 mmHg (*P*<0.001). The PaO_2_/FiO_2_ ratio also decreased markedly across respiratory support groups, with mean values of 404.3 ± 46.4, 207.0 ± 50.6, and 78.9 ± 34.2, respectively (*P* < 0.001). Respiratory rate and pH also differed significantly across groups (both *P* < 0.001).

**Table 2:** Comparison of LUS score and physiological parameters across respiratory support groups.

**O**_**2**_ **level**	**N**	**Parameter**	**Mean ± SD**
No oxygen support	85	LUS Score	0.5 ± 2.1
Low-flow oxygen support	164	LUS Score	15.0 ± 6.4
Escalated respiratory support	18	LUS Score	27.0 ± 4.8
No oxygen support	85	PaO2 (mmHg)	84.9 ± 9.7
Low-flow oxygen support	164	PaO2 (mmHg)	55.4 ± 12.7
Escalated respiratory support	18	PaO2 (mmHg)	36.7 ± 11
No oxygen support	85	PaCO2 (mmHg)	33.2 ± 6.9
Low-flow oxygen support	164	PaCO2 (mmHg)	38.4 ± 9.5
Escalated respiratory support	18	PaCO2 (mmHg)	55.2 ± 13.3
No oxygen support	85	PaO_2_/FiO_2_ (mmHg)	404.3 ± 46.4
Low-flow oxygen support	164	PaO_2_/FiO_2_ (mmHg)	207.0 ± 50.6
Escalated respiratory support	18	PaO_2_/FiO_2_ (mmHg)	78.9 ± 34.2

**Notes.**

*P* values:LUS score, *P* < 0.001; PaO_2_, *P* < 0.001; PaCO_2_, *P* < 0.001; PaO_2_/FiO_2_, *P* < 0.001; pH, *P* < 0.001; respiratory rate, *P* < 0.001.

All values are presented as mean ± SD.

### Correlations between LUS score and physiological variables

Strong correlations were observed between the LUS score and physiological markers (Table 3, Figs. 2A–2B). The LUS score showed a strong negative correlation with PaO_2_ (Spearman’s *ρ* = −0.705, *P* < 0.001), a moderate positive correlation with PaCO_2_ (*ρ* = 0.399, *P* < 0.001), a moderate negative correlation with pH (*ρ* = −0.405, *P* < 0.001), a moderate positive correlation with respiratory rate (*ρ* = 0.426, *P* < 0.001), and a strong negative correlation with the PaO_2_/FiO_2_ ratio (*ρ* = −0.764, *P* < 0.001). These parameters were in turn strongly correlated with respiratory support level, most notably PaO_2_ (*ρ* = −0.783, *P* < 0.001), PaO_2_/FiO_2_ (*ρ* = −0.848, *P* < 0.001), and respiratory rate (*ρ* = 0.386, *P* < 0.001).

**Table 3:** Spearman rank correlations of physiological parameters with LUS score and respiratory support level.

	**Correlation with LUS score**		**Correlation with oxygen therapy level**	
**Variable**	**Spearman *ρ***	***P*** **_value**	**Spearman *ρ***	***P*** **_value**
PaO2	−0.705	<0.001	−0.783	<0.001
PaCO2	0.399	<0.001	0.377	<0.001
Respiratory Rate	0.426	<0.001	0.386	<0.001
pH	−0.405	<0.001	−0.405	<0.001
PaO_2_/FiO_2_	−0.764	<0.001	−0.848	<0.001

**Notes.**

LUS, lung ultrasound; PaO_2_, arterial partial pressure of oxygen; PaCO_2_, arterial partial pressure of carbon dioxide; respiratory support level was coded as 1 = no oxygen support, 2 = low-flow oxygen support, and 3 = escalated respiratory support.

**Table 4:** ROC analysis for predicting respiratory support requirement: unadjusted and adjusted models.

Outcome	Model	AUC	AUC_95CI	Cutoff	Sensitivity	Specificity	PPV	NPV	Accuracy
Any O2 requirement	LUS score (Unadjusted)	0.982	0.968–0.997	5.5	92.3	97.6	98.8	85.6	94
Any O2 requirement	LUS score (Adjusted)	0.985	0.971–0.999	–	96.7	95.3	97.8	93.1	96.3
Any O2 requirement	PaO2 (Unadjusted)	0.961	0.937–0.985	–	88.5	97.6	98.8	79.8	91.4
Any O2 requirement	PaCO2 (Unadjusted)	0.688	0.624–0.751	–	40.7	91.8	91.4	41.9	56.9
Any O2 requirement	Full Model (LUS+ Blood gas+Covariates)	0.996	0.991–1.000	–	97.8	97.6	98.9	95.4	97.8
Escalated respiratory support	LUS Score	0.965	0.917–1.000	22.5	94.4	94.4	NA	NA	NA

**Notes.**

Cutoff determined by Youden index. PPV, positive predictive value; NPV, negative predictive value.

Adjusted models control for: gestational age, birth weight, sex, delivery mode, and Apgar score.

DeLong test comparing LUS unadjusted vs adjusted: *P* = 0.375 (no significant difference).

**Figure 3:**
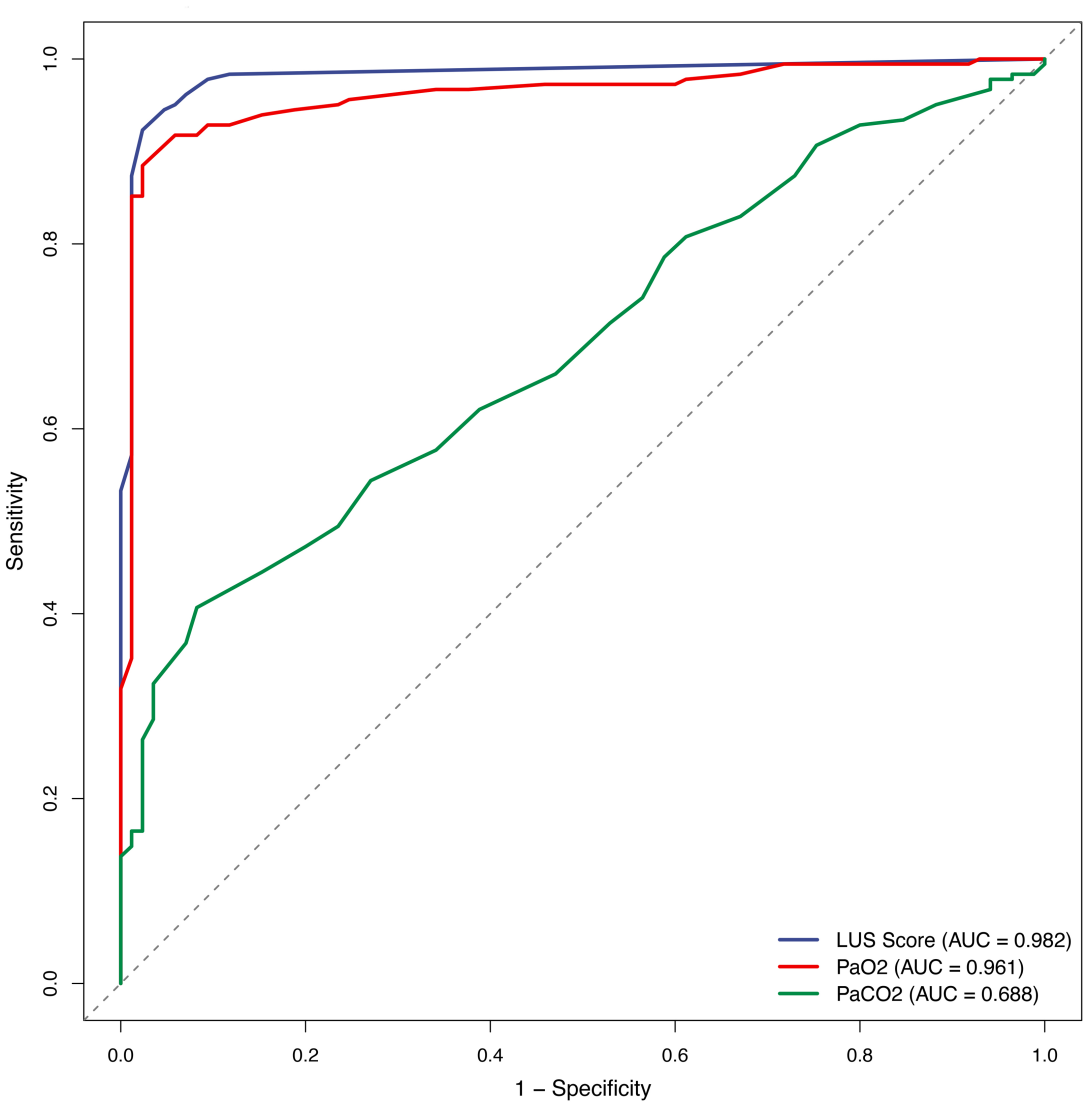
ROC curves of LUS score for predicting respiratory support requirement.

### Diagnostic accuracy for predicting oxygen therapy

ROC analysis demonstrated excellent predictive performance (Table 4, Fig. 3). For predicting any oxygen requirement (Level ≥1), LUS score achieved AUC =0.982 (95% CI [0.968–0.997]), superior to PaO_2_ (AUC = 0.961, 95% CI [0.937–0.985], DeLong *P* = 0.03) and PaCO_2_ (AUC = 0.688, 95% CI [0.624–0.751], DeLong *P* < 0.001).

The optimal LUS cutoff was 5.5, providing sensitivity 92.3%, specificity 97.6%, PPV 98.8%, and NPV 85.6%. For predicting high-flow oxygen (Level ≥ 2), LUS score achieved AUC= 0.965 (95% CI [0.917−1.000]), with optimal cutoff of 22.5 providing sensitivity and specificity both 94.4%. Among the 18 infants in the escalated respiratory support group, 5 received high-flow nasal cannula, 11 received CPAP, and 2 required invasive mechanical ventilation.

### Independent predictors of respiratory support severity

**Figure 4:**
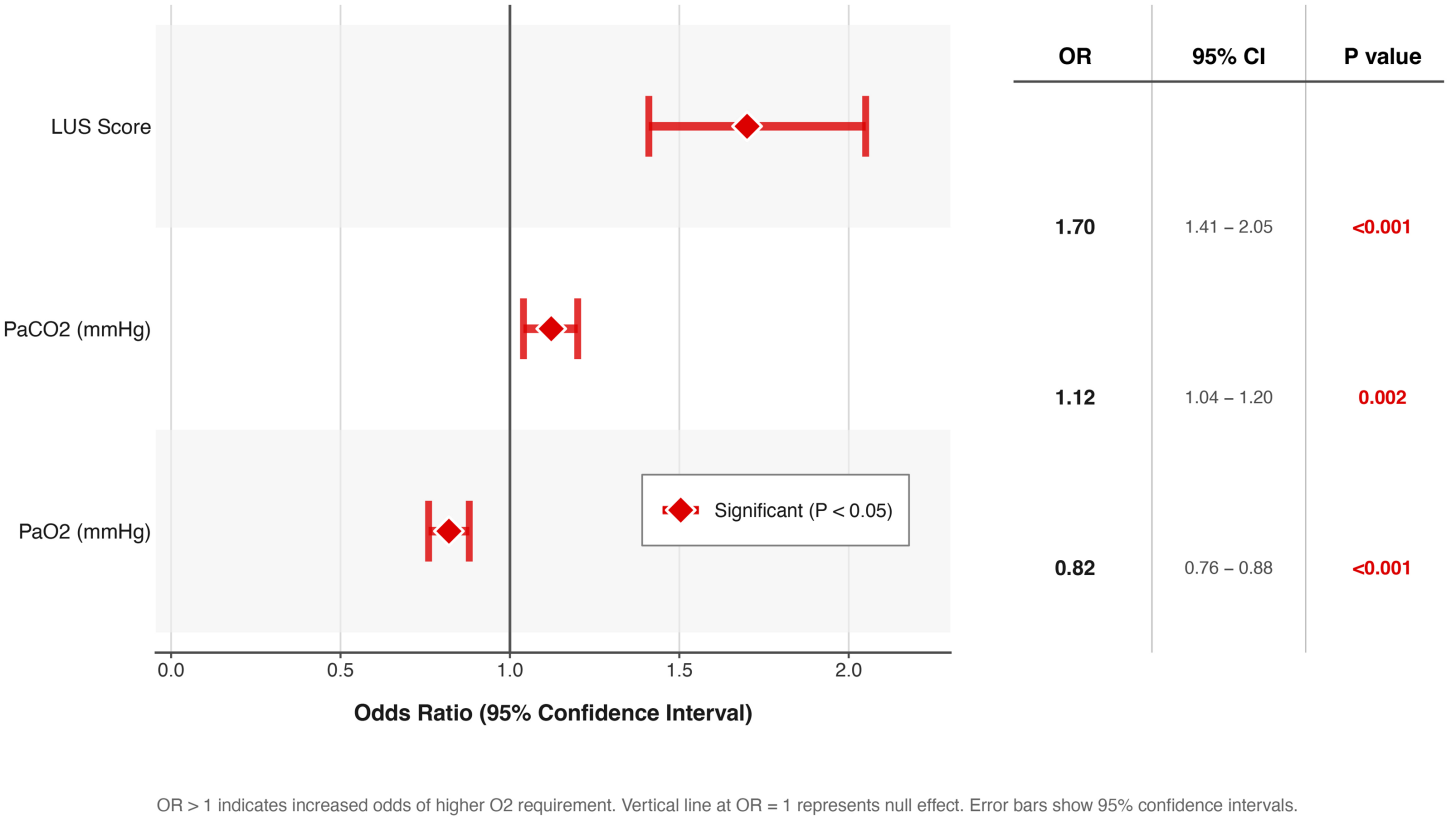
Multivariable ordinal logistic regression for predictors of respiratory support escalation.

In multivariable ordinal logistic regression (Table 5, Fig. 4), LUS score remained a highly significant independent predictor after adjusting for PaO_2_, PaCO_2_, gestational age, and delivery mode (aOR =1.700, 95% CI [1.41–2.05], *P* < 0.001), indicating each 1-point increase in LUS score increased odds of requiring higher respiratory support level by 70%. PaO_2_ was independently protective (aOR = 0.820, 95% CI [0.76–0.88], *P* < 0.001), and PaCO_2_ was an independent risk factor (aOR = 1.122, 95% CI [1.04–1.20], *P* = 0.002). Gestational age (*P* = 0.205) and delivery mode (*P* = 0.918) were not significant independent predictors.

**Table 5:** Multivariable ordinal logistic regression for O2 therapy level.

**Variable**	**Wald *χ*^2^**	**df**	**P value**	**OR**	**95% CI**
LUS Score	30.762	1	<0.001	1.700	1.41–2.05
PaO2	26.178	1	<0.001	0.820	0.76–0.88
PaCO2	9.941	1	0.002	1.122	1.04–1.20
Gestational age weeks	1.604	1	0.205	—	—
Delivery Mode	0.011	1	0.918	—	—
TOTAL	44.644	5	<0.001	—	—

### Model performance and clinical utility

**Figure 5:**
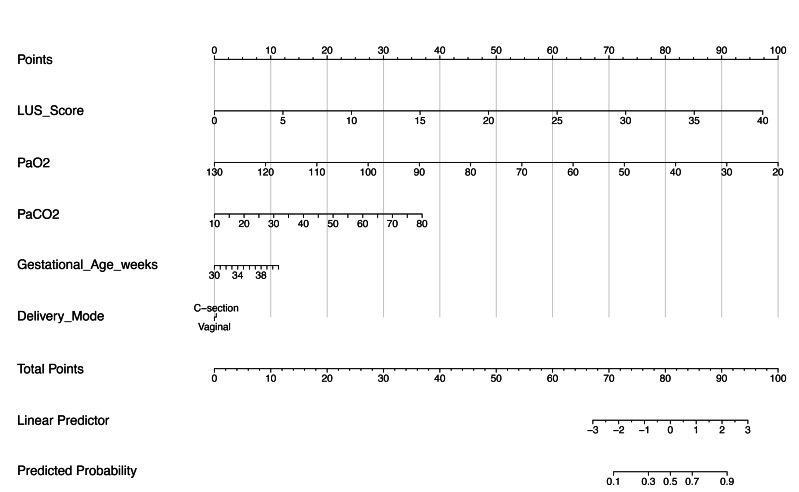
Nomogram for estimating the probability of higher respiratory support requirement.

**Figure 6:**
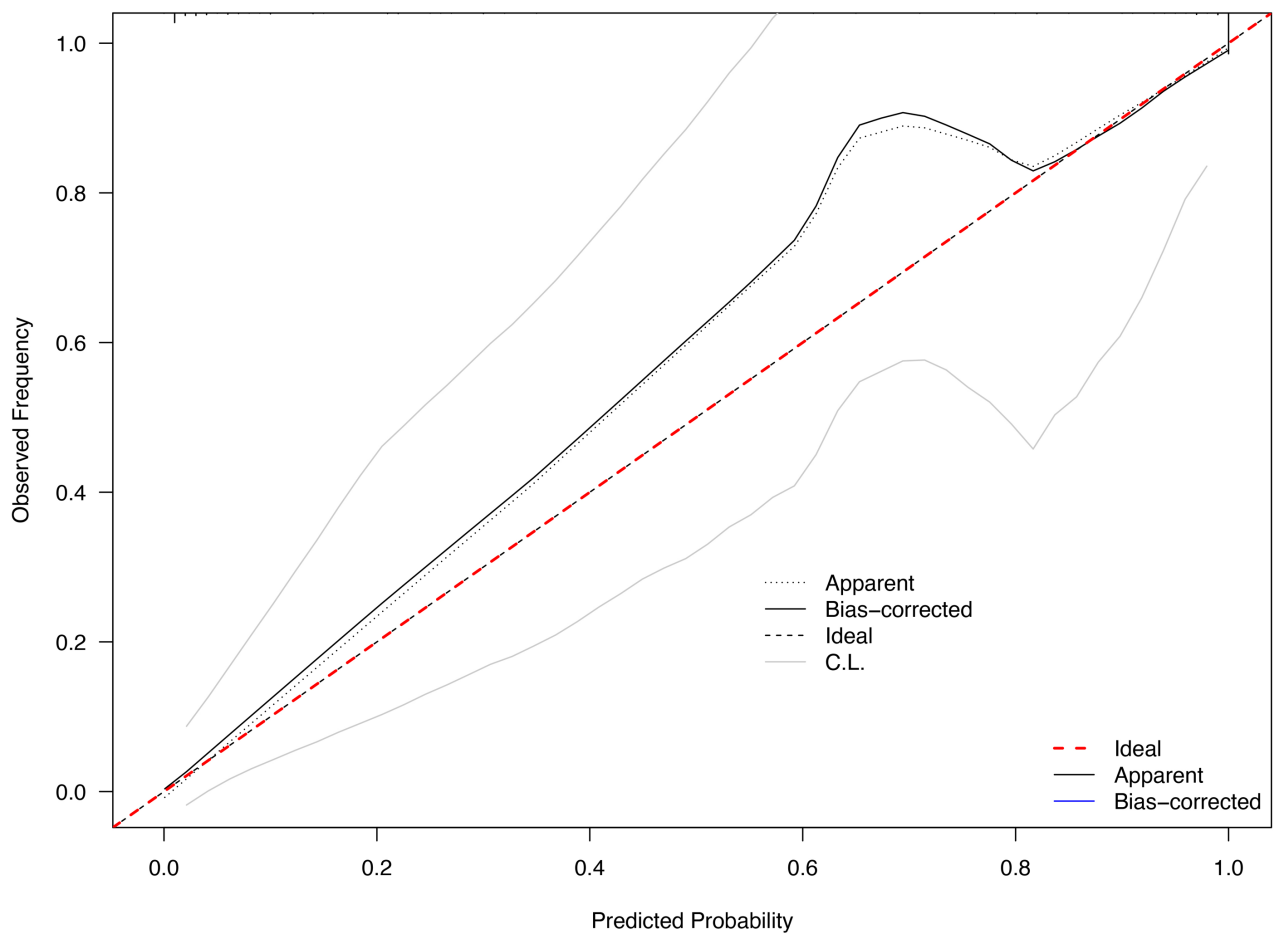
Calibration of the ordinal logistic regression model.

The prediction model was visualized as a nomogram (Fig. 5) for bedside risk estimation. Bootstrap validation (200 repetitions) demonstrated excellent calibration (Fig. 6). Decision Curve Analysis showed that the LUS score model provided superior net benefit compared to treat all or treat none strategies across threshold probabilities of 10–70% (Fig. S1, Table S2). The clinical impact curve confirmed favorable efficiency (Fig. S2).

### Subgroup and sensitivity analyses

LUS score maintained excellent accuracy in preterm neonates (*n* = 183, AUC = 0.979) and term neonates (*n* = 84, AUC = 0.948), as well as in males (*n* = 152, AUC = 0.977) and females (*n* = 115, AUC = 0.990) (Table S1). Sensitivity analyses confirmed that LUS score remained highly significant across unadjusted (OR = 1.95, 95% CI [1.59–2.38]), standard-adjusted (OR = 1.95, 95% CI [1.54–2.46]), and fully-adjusted models (OR = 1.97, 95% CI [1.55–2.50]) (Table S3). Adjusting for covariates did not significantly alter discrimination (unadjusted AUC 0.982 *vs* adjusted AUC 0.986, DeLong *P* = 0.375).

## Discussion

In this study, we demonstrate that a semi-quantitative lung ultrasound B-line score is a powerful, non-invasive tool for grading the severity of Transient Tachypnea of the Neonate (TTN). Our findings support the hypothesis that the sonographic burden of B-lines reflects the degree of lung aeration loss in TTN, which correlates with gas exchange impairment. However, B-lines are not specific to extravascular lung water alone; they are ultrasound artifacts that may also be influenced by other subpleural and interstitial changes. In TTN, where delayed fetal lung fluid clearance is the dominant mechanism, the LUS score is best interpreted as a marker of aeration loss and disease severity rather than a direct quantitative measure of lung water.

The strong correlations observed between the LUS score and objective physiological markers provide a pathophysiological basis for its predictive utility. TTN is characterized by delayed resorption of lung fluid. In TTN, increased B-line density is biologically consistent with reduced lung aeration associated with delayed fetal lung fluid clearance, which in turn is accompanied by impaired gas exchang (Gargani & Volpicelli, 2014; Kelner et al., 2026; Volpicelli et al., 2012). Our data confirms that as the sonographic burden of B-lines increases, there is a commensurate deterioration in oxygenation (PaO_2_) and ventilation (PaCO_2_). This relationship supports the LUS score as a clinically useful marker of physiological impairment in TTN, extending prior observations that linked LUS patterns to oxygenation indices (Brat et al., 2015; Razak & Faden, 2020). Unlike static chest radiography, which correlates poorly with functional severity (Perri et al., 2018), the LUS score dynamically reflects the magnitude of interstitial edema, offering a more accurate assessment of the underlying pathology.

A critical clinical contribution of this study is the definition of pragmatic thresholds for TTN management. The identification of a lower threshold (score > 5.5) provides a highly sensitive screening tool to distinguish neonates requiring oxygen therapy from those suitable for observational management. Conversely, the higher threshold (score > 22.5) serves as an early warning signal for the potential need to escalate respiratory support beyond low-flow oxygen, including HFNC or non-invasive ventilation. While previous studies have proposed cutoffs for varying outcomes (Brat et al., 2015; Vergine et al., 2014), such as predicting CPAP failure in NRDS, our study extends prior work by defining separate thresholds for any oxygen support *versus* escalated respiratory support in a cohort of neonates with TTN. This offers a more granular approach to management, allowing for a stepwise escalation of care. The Level 2 outcome should therefore be interpreted as need for escalation beyond low-flow oxygen rather than as equivalence among specific advanced respiratory support modalities.

Furthermore, the robustness of the LUS score was confirmed through multivariable analysis. A potential concern in observational studies is that the severity of respiratory distress may simply reflect underlying factors such as lower gestational age or delivery mode. However, our model demonstrated that the LUS score remained the strongest predictor of oxygen therapy escalation even after adjusting for these confounders and physiological variables. This independence suggests that the LUS score captures unique anatomical information, specifically the extent of lung aeration loss, that is not fully represented by demographic factors or systemic blood gas values alone (Hiles et al., 2017). The Decision Curve Analysis reinforces this finding, showing that LUS-guided decision-making yields a higher net clinical benefit than relying solely on clinical judgement or treating all patients, thereby supporting its integration into standard protocols to optimize resource allocation. It should be noted that because blood gas values are routinely used by clinicians to guide respiratory support decisions, the comparison between LUS score and blood gas parameters as predictors of the same outcome is potentially affected by incorporation bias. Accordingly, these comparisons should be interpreted with caution.

The clinical impact of our findings extends beyond immediate oxygen therapy decisions. Recent multicenter studies have demonstrated that LUS scoring can predict long-term respiratory outcomes, including bronchopulmonary dysplasia (Liu et al., 2016), and guide surfactant replacement therapy in extremely preterm neonates (De Martino et al., 2018; Rodriguez-Fanjul et al., 2020). Our decision curve analysis framework aligns with emerging efforts to integrate LUS into standardized clinical algorithms (Raimondi et al., 2012), supporting a paradigm shift toward precision respiratory care in the NICU.

Our study has several limitations that warrant consideration. First, as a single-center study, the generalizability of the specific cutoffs to other institutions with different equipment or operator experience levels requires validation. Although discrimination was high, these estimates may be optimistic because score derivation and evaluation were performed in a single center with standardized operators and local clinical pathways. The proposed thresholds should therefore be regarded as internally derived candidate cutoffs requiring external validation in other settings before clinical adoption. Second, although all operators were trained according to established guidelines (Kurepa et al., 2018), formal inter-observer reliability of the LUS score was not prospectively assessed in this cohort. While prior multicenter studies suggest reasonable reproducibility of neonatal LUS findings when standardized protocols are applied (Raimondi et al., 2019), blinded duplicate scoring in future studies would strengthen the evidence for the score’s reproducibility. In addition, standardized clinical respiratory distress scores, such as the Silverman-Andersen Respiratory Severity Score, were not incorporated into the present analysis. Future studies evaluating the complementary roles of LUS scoring and established clinical severity scores may further improve characterization of disease severity in TTN. Third, the sample size for the escalated respiratory support group was relatively small compared with the other groups; while the statistical significance was strong, larger multicenter cohorts are needed to refine the precision of the higher-severity threshold. Baseline differences between severity groups, including lower gestational age and birth weight in infants requiring greater support, may have contributed to the observed differences in LUS scores. Although multivariable adjustment was performed, residual confounding by unmeasured factors cannot be excluded. Finally, this study focused on the immediate need for respiratory support and did not evaluate long-term pulmonary outcomes. We acknowledge that some diagnostic overlap between severe TTN and mild NRDS is inherent in the early postnatal period, and we cannot completely exclude that a small number of infants in the escalated-support group may have had an element of surfactant deficiency.

In practice, a global LUS score below 6 may support observation in room air, whereas scores above 22.5 should prompt close monitoring and a low threshold for initiating high-flow support or CPAP. These thresholds could be incorporated into standardized NICU pathways for early respiratory assessment. Future multicenter studies are needed to confirm these cutoffs and to evaluate whether LUS-guided strategies improve clinical outcomes and resource use. Incorporation of additional physiological indices, such as ventilatory ratio, may further improve understanding of the relationship between lung aeration and respiratory function. However, the LUS score should not be interpreted as a standalone predictor of the need for invasive mechanical ventilation. Decisions regarding CPAP initiation or escalation to invasive ventilation require comprehensive clinical assessment, including evaluation of work of breathing, hemodynamic stability, oxygenation trajectory, and response to initial support. Advanced ventilatory indices such as ventilatory ratio and dead-space fraction were not available in our cohort, as most infants were spontaneously breathing or receiving noninvasive support and these parameters were not routinely measured in our NICU for this population.

## Conclusions

The LUS B-line score is a valid, physiologically coherent tool that can help grade the severity of TTN and stratify early oxygen and respiratory support needs at the bedside. By semi-quantitatively reflecting the degree of lung aeration loss, the LUS score provides candidate thresholds (5.5 and 22.5) that may assist in decisions regarding the initiation and escalation of respiratory support. However, decisions regarding CPAP or invasive mechanical ventilation should continue to rely on comprehensive clinical assessment. These thresholds require external validation before routine clinical adoption.

## Supplemental Information

10.7717/peerj.21559/supp-1 Supplemental Information 1 Subgroup analysis of the diagnostic performance of the LUS score for predicting respiratory support requirement

10.7717/peerj.21559/supp-2 Supplemental Information 2 Net Benefit at Key Threshold Probabilities.

10.7717/peerj.21559/supp-3 Supplemental Information 3 Comparison of Unadjusted and Adjusted ROC Analyses

10.7717/peerj.21559/supp-4 Supplemental Information 4 Decision Curve Analysis evaluating the clinical utility of the LUS score-based prediction model

10.7717/peerj.21559/supp-5 Supplemental Information 5 Clinical Impact Curve showing the estimated number of high-risk patients versus true positives

10.7717/peerj.21559/supp-6 Supplemental Information 6 Raw data of lung ultrasound B-line scoring. Distribution of each variable

10.7717/peerj.21559/supp-7 Supplemental Information 7 Codebook.

10.7717/peerj.21559/supp-8 Supplemental Information 8 STROBE checklist.

## Additional Information and Declarations

### Data Availability

The following information was supplied regarding data availability: The raw measurements are available in the Supplemental Files.
