# Supplementary material for: A lung ultrasound B-line score to stratify oxygen therapy in transient tachypnea of the neonate: a prospective cohort study
Source: PeerJ. 2026 Jul 22;14:e21559. doi: 10.7717/peerj.21559 (PMC13401361; doi:10.7717/peerj.21559)
Supplement: Supplemental Information 1 [file peerj-14-21559-s001.docx]

**Supplementary Table 1. Subgroup Analysis of LUS Score for Predicting Oxygen Therapy Requirement**

| **Category** | **Subgroup** | **N** | **No O2** | **Need O2** | **AUC** | **95% CI** | **Cutoff** | **Sens (%)** | **Spec (%)** |
| --- | --- | --- | --- | --- | --- | --- | --- | --- | --- |
| Gestational Age | Preterm | 183 | 31 | 152 | 0.979 | 0.952 - 1 | 5.5 | 94.7 | 93.5 |
|  | Term | 84 | 54 | 30 | 0.948 | 0.891 - 1 | 2.0 | 86.7 | 100.0 |
| Sex | Male | 152 | 49 | 103 | 0.977 | 0.953 - 1 | 5.5 | 90.3 | 98.0 |
|  | Female | 115 | 36 | 79 | 0.990 | 0.976 - 1 | 5.5 | 94.9 | 97.2 |
| Delivery Mode | C-section | 79 | 32 | 47 | 0.995 | 0.986 - 1 | 5.5 | 93.6 | 100.0 |
|  | Vaginal | 188 | 53 | 135 | 0.977 | 0.956 - 0.999 | 4.5 | 94.8 | 96.2 |
| AUC: Area Under the Curve. Cutoff determined by Youden index. Sens: Sensitivity, Spec: Specificity. Green: AUC ≥ 0.9 (excellent); Yellow: 0.8 ≤ AUC < 0.9 (good). | | | | | | | | | |
