# Supplementary material for: A lung ultrasound B-line score to stratify oxygen therapy in transient tachypnea of the neonate: a prospective cohort study
Source: PeerJ. 2026 Jul 22;14:e21559. doi: 10.7717/peerj.21559 (PMC13401361; doi:10.7717/peerj.21559)
Supplement: Supplemental Information 2 [file peerj-14-21559-s002.docx]

**Supplementary Table 2.** Subgroup analysis of the diagnostic performance of the LUS score for predicting respiratory support requirement.

| **Threshold Probability** | **Treat All Strategy** | **LUS Score Model** | **PaO2 Model** | **LUS + PaO2 Model** | **Full Model** |
| --- | --- | --- | --- | --- | --- |
| 10% | 0.6463 | 0.6663 | 0.6417 | 0.6733 | 0.6767 |
| 20% | 0.6021 | 0.6592 | 0.6292 | 0.6723 | 0.6685 |
| 30% | 0.5452 | 0.6458 | 0.6180 | 0.6602 | 0.6640 |
| 40% | 0.4694 | 0.6355 | 0.6042 | 0.6592 | 0.6567 |
| 50% | 0.3633 | 0.6292 | 0.5955 | 0.6517 | 0.6517 |
| Net benefit = (True Positive Rate) - (False Positive Rate) × [pt/(1-pt)] | | | | | |
| where pt is the threshold probability. | | | | | |
| Higher values indicate better clinical utility. | | | | | |
| Treat All strategy assumes all patients receive intervention. | | | | | |
