# Supplementary material for: A lung ultrasound B-line score to stratify oxygen therapy in transient tachypnea of the neonate: a prospective cohort study
Source: PeerJ. 2026 Jul 22;14:e21559. doi: 10.7717/peerj.21559 (PMC13401361; doi:10.7717/peerj.21559)
Supplement: Supplemental Information 3 [file peerj-14-21559-s003.docx]

Supplementary Table 3. Comparison of Unadjusted and Adjusted ROC Analyses

| **Model** | **AUC (95% CI)** | **Cutoff** | **Sensitivity** | **Specificity** |
| --- | --- | --- | --- | --- |
| LUS Score (Unadjusted) | 0.982 (0.968 - 0.997) | 0.77 | 92.3 | 97.6 |
| LUS Score (Adjusted for Covariates) | 0.986 (0.973 - 0.999) | 0.37 | 96.7 | 95.3 |
| Full Model (LUS + Blood Gas + Covariates) | 0.996 (0.991 - 1.000) | 0.51 | 97.8 | 97.6 |
| Adjusted models control for gestational age, weight, sex, delivery mode, and Apgar score at 5 min. | | | | |
