# Supplementary figures and images for: A lung ultrasound B-line score to stratify oxygen therapy in transient tachypnea of the neonate: a prospective cohort study
Source: PeerJ. 2026 Jul 22;14:e21559. doi: 10.7717/peerj.21559 (PMC13401361; doi:10.7717/peerj.21559)

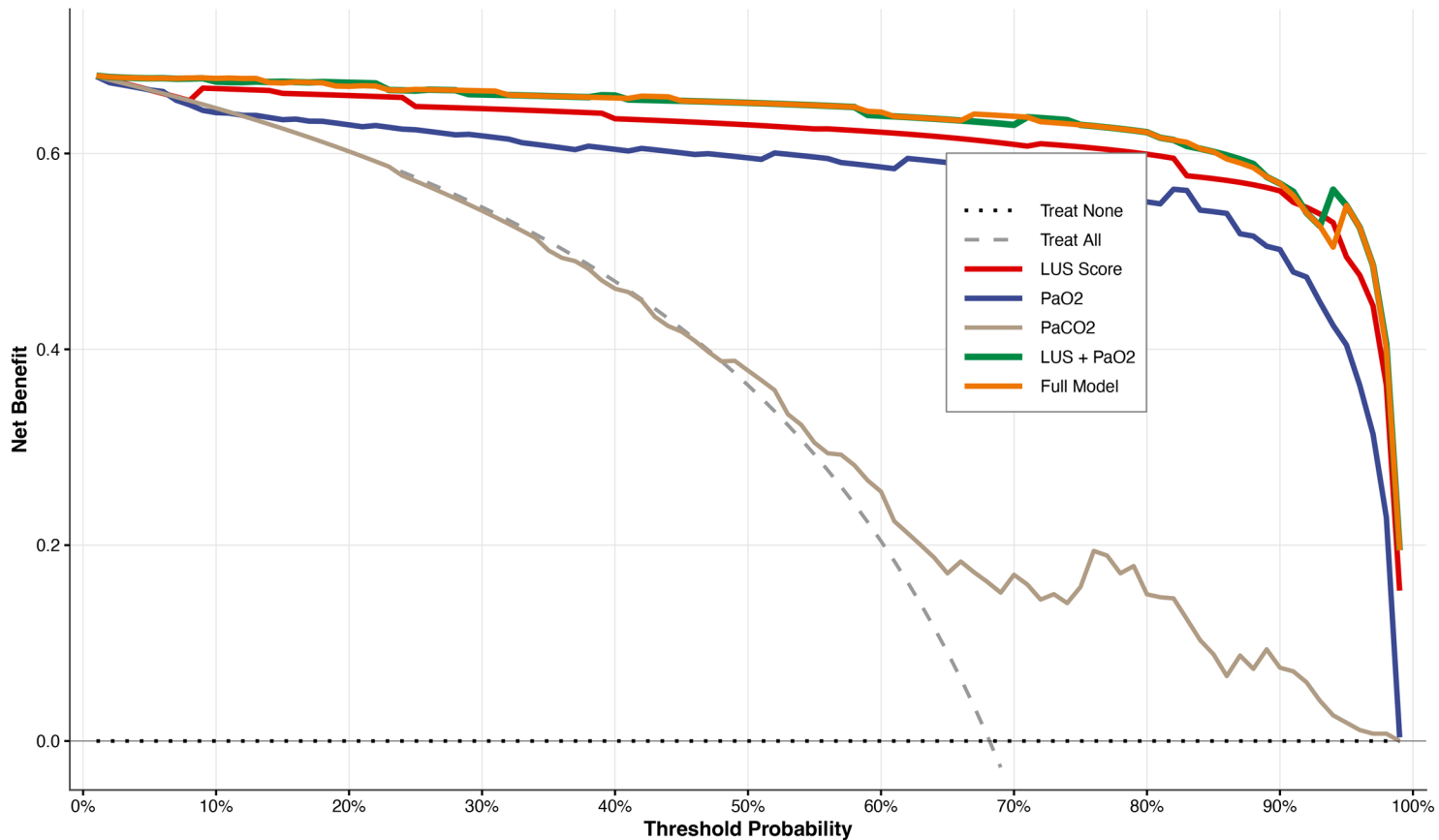

Higher net benefit indicates better clinical utility for decision-making

Supplement: Supplemental Information 4 [file peerj-14-21559-s004.pdf]

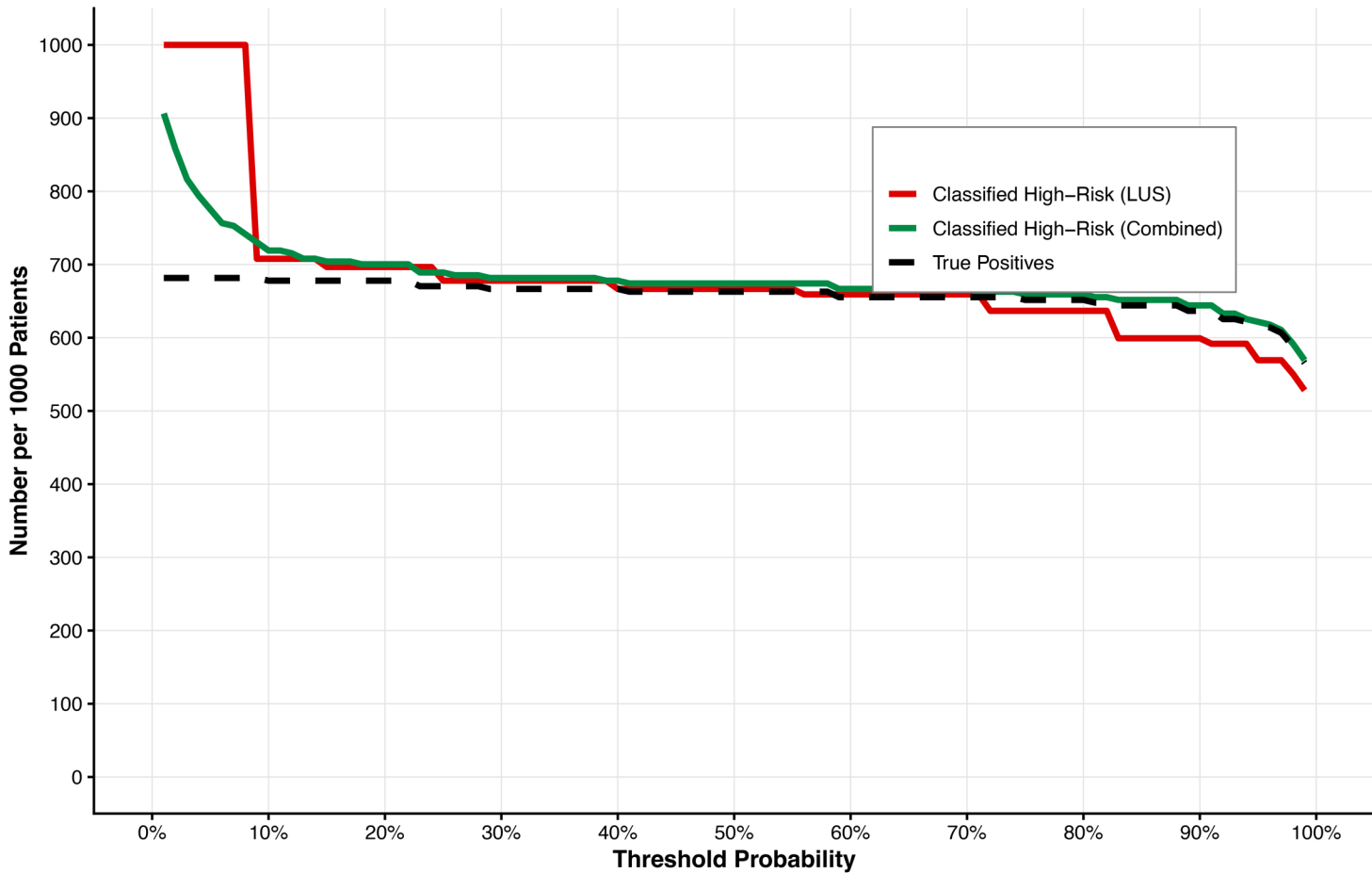

Supplement: Supplemental Information 5 [file peerj-14-21559-s005.pdf]
