# Supplementary material for: A lung ultrasound B-line score to stratify oxygen therapy in transient tachypnea of the neonate: a prospective cohort study
Source: PeerJ. 2026 Jul 22;14:e21559. doi: 10.7717/peerj.21559 (PMC13401361; doi:10.7717/peerj.21559)
Supplement: Supplemental Information 8 [file peerj-14-21559-s008.doc]

STROBE Statement—Checklist of items that should be included in reports of ***cohort studies***

|  | Item No | Recommendation | Corresponding Information/Description from the Manuscript |
| --- | --- | --- | --- |
| **Title and abstract** | 1 | (*a*) Indicate the study’s design with a commonly used term in the title or the abstract | Yes. The title and abstract explicitly state it is a "Prospective Cohort Study |
|  |  | (*b*) Provide in the abstract an informative and balanced summary of what was done and what was found | Yes. The Abstract includes the Background, Methods (design, enrollment, LUS score, outcome), Results (correlations, AUCs, aORs), and Conclusions. |
| Introduction | | |  |
| Background/rationale | 2 | Explain the scientific background and rationale for the investigation being reported | Yes. The rationale discusses TTN as the most common cause of neonatal respiratory distress, the limitations of current methods (clinical scores are subjective, CXR is insensitive), and the hypothesis that a quantitative LUS B-line score may reflect lung fluid volume. |
| Objectives | 3 | State specific objectives, including any prespecified hypotheses | Yes. The primary objectives were to quantify the relationship between the LUS score and arterial oxygenation ($PaO_2$) and ventilation ($PaCO_2$), and to determine precise score thresholds that discriminate between neonates requiring no respiratory support, low-flow oxygen, and high-flow interventions |
| Methods | | |  |
| Study design | 4 | Present key elements of study design early in the paper | Yes. Stated as a "prospective observational study" |
| Setting | 5 | Describe the setting, locations, and relevant dates, including periods of recruitment, exposure, follow-up, and data collection | Yes. Conducted in the Level III Neonatal Intensive Care Unit at Chongqing University Fuling Hospital, from January 1, 2022, to September 30, 2024 |
| Participants | 6 | (*a*) Give the eligibility criteria, and the sources and methods of selection of participants. Describe methods of follow-up | Yes. Eligibility criteria included neonates with gestational age ≥33 weeks and birth weight ≥1800 g, admitted within 24 hours of birth with symptom onset within the first 6 hours, and a clinical diagnosis of TTN7. Exclusion criteria are listed8. The primary outcome was assessed within 72 hours |
|  |  | (*b*) For matched studies, give matching criteria and number of exposed and unexposed | N/A. This was not a matched study. |
| Variables | 7 | Clearly define all outcomes, exposures, predictors, potential confounders, and effect modifiers. Give diagnostic criteria, if applicable | Yes. Predictor: Standardized twelve-zone LUS B-line score (range 0–36). Primary Outcome: Maximum oxygen therapy level within 72 hours, classified as Level 0, Level 1, or Level 2. Potential Confounders: Gestational age, mode of delivery, PaO2, and PaCO2 |
| Data sources/ measurement | 8* | For each variable of interest, give sources of data and details of methods of assessment (measurement). Describe comparability of assessment methods if there is more than one group | Yes. LUS was performed within 6 hours of admission using a high-frequency linear transducer (8–12 MHz) and a standardized twelve-zone protocol. Scoring details (0 to 3 per zone) are provided. Arterial Blood Gas (ABG) analysis was performed via radial artery puncture or indwelling catheter within 6 hours. |
| Bias | 9 | Describe any efforts to address potential sources of bias | Yes. Efforts to minimize bias included blinding the LUS operators to ABG results and clinical severity grading , and blinding the attending neonatologists making the respiratory support decisions to the LUS scores |
| Study size | 10 | Explain how the study size was arrived at | Yes. It was estimated that a sample size of 240 infants would provide 90% power to detect an AUC of 0.85 or greater, assuming a prevalence of oxygen requirement of 60%. |
| Quantitative variables | 11 | Explain how quantitative variables were handled in the analyses. If applicable, describe which groupings were chosen and why | Yes. Continuous variables were expressed as means ± standard deviations (SD) or medians with interquartile ranges (IQR)19. LUS score was used as a continuous variable in correlation and regression, and specific cutoffs (5.5 and 22.5) were derived using the Youden index for dichotomous prediction. |
| Statistical methods | 12 | (*a*) Describe all statistical methods, including those used to control for confounding | Yes. Methods described include Kruskal-Wallis/one-way ANOVA, Chi-square test, Spearman's. Confounding was controlled using a multivariable ordinal logistic regression model. |
|  |  | (*b*) Describe any methods used to examine subgroups and interactions | Yes. Subgroup analyses were described for preterm vs. term neonates, and male vs. female infants. |
|  |  | (*c*) Explain how missing data were addressed | Not explicitly stated. The manuscript does not detail the handling of missing data |
|  |  | (*d*) If applicable, explain how loss to follow-up was addressed | N/A. Due to the short follow-up time (72 hours) and NICU setting, no loss to follow-up was reported. |
|  |  | (*e*) Describe any sensitivity analyses | Yes. Sensitivity analyses were performed to compare unadjusted and adjusted models (standard-adjusted and fully-adjusted) for predicting oxygen therapy escalation |
| Results | | |  |
| Participants | 13* | (a) Report numbers of individuals at each stage of study—eg numbers potentially eligible, examined for eligibility, confirmed eligible, included in the study, completing follow-up, and analysed | Yes. A total of 267 neonates were included in the final analysis. Numbers by outcome group are provided: No O2 (n=85), Low-Flow O2 (n=164), and High-Flow O2 (n=18). |
|  |  | (b) Give reasons for non-participation at each stage | No. Specific numbers and reasons for non-participation/exclusion are not provided. |
|  |  | (c) Consider use of a flow diagram | No. A flow diagram is not explicitly mentioned or included in the legends |
| Descriptive data | 14* | (a) Give characteristics of study participants (eg demographic, clinical, social) and information on exposures and potential confounders | Yes. Baseline characteristics, including gestational age, birth weight, Apgar scores, and term neonate proportion, are provided stratified by oxygen group |
|  |  | (b) Indicate number of participants with missing data for each variable of interest | No. The number of participants with missing data for individual variables is not explicitly stated. |
|  |  | (c) Summarise follow-up time (eg, average and total amount) | Yes. The primary outcome was assessed over a follow-up period of 72 hours. |
| Outcome data | 15* | Report numbers of outcome events or summary measures over time | Yes. The number of patients in each outcome level is reported (85, 164, 18). Summary measures like median ventilation duration and median length of stay are also provided across groups |
| Main results | 16 | (*a*) Give unadjusted estimates and, if applicable, confounder-adjusted estimates and their precision (eg, 95% confidence interval). Make clear which confounders were adjusted for and why they were included | Yes. Unadjusted Spearman's for correlations is provided30. Adjusted estimates from multivariable ordinal logistic regression (aOR = 1.70; 95% CI, 1.41–2.05) are provided, adjusted for PaO2, PaCO2, gestational age, and delivery mode |
|  |  | (*b*) Report category boundaries when continuous variables were categorized | Yes. Optimal LUS score cutoffs of 5.5 and 22.5 are reported for predicting oxygen requirement and high-flow support, respectively. |
|  |  | (*c*) If relevant, consider translating estimates of relative risk into absolute risk for a meaningful time period | No. Relative risk was not used; absolute risk was not directly calculated, though Decision Curve Analysis (DCA) provided net benefit based on threshold probability |
| Other analyses | 17 | Report other analyses done—eg analyses of subgroups and interactions, and sensitivity analyses | Yes. Subgroup analyses are reported, confirming accuracy in preterm vs. term neonates, and males vs. females. Sensitivity analyses comparing unadjusted and adjusted models are also summarized. |
| Discussion | | |  |
| Key results | 18 | Summarise key results with reference to study objectives | Yes. Key results on the strong correlation between LUS score and gas exchange (PaO2, PaCO2) are summarized, and the clinical utility of the specific cutoffs (5.5 and 22.5) is highlighted. |
| Limitations | 19 | Discuss limitations of the study, taking into account sources of potential bias or imprecision. Discuss both direction and magnitude of any potential bias | Yes. Limitations discussed include: single-center design (generalizability), lack of formal inter-observer variability assessment, and the small sample size for the high-flow group |
| Interpretation | 20 | Give a cautious overall interpretation of results considering objectives, limitations, multiplicity of analyses, results from similar studies, and other relevant evidence | Yes. The discussion provides pathophysiological interpretation (LUS score as a surrogate for lung fluid) and interprets the results in the context of multivariable analysis (LUS score's independence from confounders) and existing literature. |
| Generalisability | 21 | Discuss the generalisability (external validity) of the study results | Yes. The discussion explicitly mentions that the specific cutoffs may require validation in other institutions due to the single-center nature of the study. |
| Other information | | |  |
| Funding | 22 | Give the source of funding and the role of the funders for the present study and, if applicable, for the original study on which the present article is based | Yes. The source of funding is the Science and Health Joint Medical Research Project of Fuling District, Chongqing (Grant No. 2024KWLH006). It states the funders had no role in study design, data collection/analysis, or publication decisions |

*Give information separately for exposed and unexposed groups.

**Note:** An Explanation and Elaboration article discusses each checklist item and gives methodological background and published examples of transparent reporting. The STROBE checklist is best used in conjunction with this article (freely available on the Web sites of PLoS Medicine at http://www.plosmedicine.org/, Annals of Internal Medicine at http://www.annals.org/, and Epidemiology at http://www.epidem.com/). Information on the STROBE Initiative is available at http://www.strobe-statement.org.
